# DNA Repair Gene Polymorphisms and Chromosomal Aberrations in Exposed Populations

**DOI:** 10.3389/fgene.2021.691947

**Published:** 2021-06-16

**Authors:** Yasmeen Niazi, Hauke Thomsen, Bozena Smolkova, Ludmila Vodickova, Sona Vodenkova, Michal Kroupa, Veronika Vymetalkova, Alena Kazimirova, Magdalena Barancokova, Katarina Volkovova, Marta Staruchova, Per Hoffmann, Markus M. Nöthen, Maria Dusinska, Ludovit Musak, Pavel Vodicka, Kari Hemminki, Asta Försti

**Affiliations:** ^1^Department of Molecular Genetic Epidemiology, German Cancer Research Center (DKFZ), Heidelberg, Germany; ^2^Hopp Children’s Cancer Center (KiTZ), Heidelberg, Germany; ^3^Division of Pediatric Neurooncology, German Cancer Research Center (DKFZ), German Cancer Consortium (DKTK), Heidelberg, Germany; ^4^GeneWerk GmbH, Heidelberg, Germany; ^5^Department of Molecular Oncology, Cancer Research Institute, Biomedical Research Center of the Slovak Academy of Sciences, Bratislava, Slovakia; ^6^Department of Molecular Biology of Cancer, Institute of Experimental Medicine, Czech Academy of Sciences, Prague, Czechia; ^7^First Faculty of Medicine, Institute of Biology and Medical Genetics, Charles University, Prague, Czechia; ^8^Faculty of Medicine and Biomedical Center in Pilsen, Charles University in Prague, Prague, Czecia; ^9^Department of Biology, Faculty of Medicine, Slovak Medical University, Bratislava, Slovakia; ^10^Institute of Human Genetics, School of Medicine and University Hospital Bonn, University of Bonn, Bonn, Germany; ^11^Division of Medical Genetics, Department of Biomedicine, University of Basel, Basel, Switzerland; ^12^Health Effects Laboratory, Department of Environmental Chemistry, NILU-Norwegian Institute for Air Research, Kjeller, Norway; ^13^Jessenius Faculty of Medicine, Biomedical Center Martin, Comenius University in Bratislava, Bratislava, Slovakia; ^14^Division of Cancer Epidemiology, German Cancer Research Centre (DKFZ), Heidelberg, Germany

**Keywords:** chromosomal aberrations, association study, DNA repair, exposure, polymorphism

## Abstract

DNA damage and unrepaired or insufficiently repaired DNA double-strand breaks as well as telomere shortening contribute to the formation of structural chromosomal aberrations (CAs). Non-specific CAs have been used in the monitoring of individuals exposed to potential carcinogenic chemicals and radiation. The frequency of CAs in peripheral blood lymphocytes (PBLs) has been associated with cancer risk and the association has also been found in incident cancer patients. CAs include chromosome-type aberrations (CSAs) and chromatid-type aberrations (CTAs) and their sum CAtot. In the present study, we used data from our published genome-wide association studies (GWASs) and extracted the results for 153 DNA repair genes for 607 persons who had occupational exposure to diverse harmful substances/radiation and/or personal exposure to tobacco smoking. The analyses were conducted using linear and logistic regression models to study the association of DNA repair gene polymorphisms with CAs. Considering an arbitrary cutoff level of 5 × 10^–3^, 14 loci passed the threshold, and included 7 repair pathways for CTA, 4 for CSA, and 3 for CAtot; 10 SNPs were eQTLs influencing the expression of the target repair gene. For the base excision repair pathway, the implicated genes *PARP1* and *PARP2* encode poly(ADP-ribosyl) transferases with multiple regulatory functions. *PARP1* and *PARP2* have an important role in maintaining genome stability through diverse mechanisms. Other candidate genes with known roles for CSAs included *GTF2H* (general transcription factor IIH subunits 4 and 5), Fanconi anemia pathway genes, and *PMS2*, a mismatch repair gene. The present results suggest pathways with mechanistic rationale for the formation of CAs and emphasize the need to further develop techniques for measuring individual sensitivity to genotoxic exposure.

## Introduction

Human cancers are often associated with chromosomal instability with complex numerical and structural chromosomal aberrations (CAs), which may be causative events in the process of malignant transformation (Futreal et al., 2004; Rajagopalan and Lengauer, 2004; Mitelman et al., 2007; Burrell et al., 2013). Structural CAs may be specific, such as translocations and inversions, or non-specific, such as chromatid breaks, fragmented or missing parts of chromosomes, and fusions resulting in dicentric and ring chromosomes (Bignold, 2009). The former are often recurrent and they are currently analyzed by molecular cytogenetic methods while the latter are scored by classical cytogenetic techniques, which are able to recognize chromosome-type aberrations (CSAs) and chromatid-type aberrations (CTAs) according to morphological changes (Hagmar et al., 2004). CTAs are formed due to insufficiently repaired double-strand breaks (DSBs) during the late S or G2 phase of the cell cycle (Natarajan and Palitti, 2008; Bignold, 2009; Durante et al., 2013), whereas CSAs are the result of direct DNA damage due to radiation, chemical mutagens, or shortening of telomeres during the G0/G1 phase (Albertini et al., 2000; Jones et al., 2012). Non-specific CAs have been used in the monitoring of populations occupationally exposed to potential carcinogenic chemicals and radiation and an increased frequency of CAs in peripheral blood lymphocytes (PBLs) has been associated with cancer risk and the association has also been found in incident cancer patients (Rossner et al., 2005; Vodicka et al., 2010; Vodenkova et al., 2015).

Unrepaired or insufficiently repaired DSBs, as well as telomerase dysfunction, represent the mechanistic bases for the formation of structural CAs (Natarajan and Palitti, 2008; Bignold, 2009; Durante et al., 2013; Vodicka et al., 2018; Srinivas et al., 2020). However, even other types of DNA repair pathways may contribute to CA formation as these are found in inherited syndromes manifesting DNA repair gene mutations (Rahman, 2014). Eukaryotic cells have four conserved but distinct pathways of DSB repair: non-homologous DNA end joining (NHEJ), alternate end joining (a-EJ), homologous recombination (HR), and single-strand annealing (SSA) (Sung, 2018). In non-malignant cells, the majority of DSBs are removed *via* either NHEJ or HR, with minor contribution of a-EJ and SSA. Repair *via* HR may be error-free while the three other DSB repairs are error-prone, particularly the rare a-EJ and SSA. Repair errors emerge as mutations and CAs with smaller or larger DNA sequence losses. The role of telomerase dysfunction has been emerging more recently, with growing evidence that shorter telomeres are associated with increased frequency of CAs, particularly of the CSA type (Li et al., 2013; Hemminki et al., 2015). Telomeres become shorter at each round of replication and critically shortened telomeres may be poorly end-capped and may be recognized as DSBs by repair machinery that may result as CAs (Maser and DePinho, 2002; Meeker et al., 2004; Gostissa et al., 2011; Jones and Jallepalli, 2012; Maciejowski et al., 2015). It has been shown that telomere shortening is associated with a decreased capacity to repair DSBs in multiple types of cancer (Kroupa et al., 2017).

In the present study, we used data from our published genome-wide association studies (GWASs) (Niazi et al., 2018, 2019) and extracted the results for 153 DNA repair genes to find out the association between CA frequency and DNA repair pathways. The population was occupationally exposed to diverse harmful substances/radiation and/or personally exposed to tobacco smoking. The analyses were conducted for the types of CAs (CAtot, CSAs, and CTAs) using linear and logistic regression models.

## Materials and Methods

Our cohort comprised 607 individuals recruited from the Czech and Slovak Republics. The subjects were investigated for chromosomal abnormalities in previous occupational exposure-related epidemiological studies or as regular medical monitoring in factories with exposure to genotoxic compounds. These studies involved individuals with defined exposure to small organic compounds, heavy metals, radiations, and asbestos and other mineral fibers as well as unexposed controls (Vodicka et al., 2004a,b; Dusinska et al., 2004a,b, 2012; Musak et al., 2008; Kazimirova et al., 2009). Prior to blood sampling, study subjects were informed according to the rules of Helsinki declaration and written approval was obtained. Ethics Committees of the Slovak Medical University, the Jessenius Faculty of Medicine, the Comenius University Bratislava, the Institute for Clinical and Experimental Medicine in Slovakia, and the Thomayer Hospital and the General University Hospital in the Czech Republic approved the study design.

The study population (Table 1) contained about 60% males and 40% females. All individuals included in the study were either exposed to genotoxic compounds due to their occupation and/or they were smokers. About half of the individuals (52.1%) had a history of occupational exposure to genotoxic organic compounds while 12.7% were exposed to heavy metals, mineral fibers, and low levels of radiations. All subjects filled a questionnaire listing beside the type of job and periods of exposure other exogenous factors such as smoking, radiation exposure, and dietary dispositions. About 66% of the individuals included in the study were smokers. Age of the participants ranged from 19 to 80 years with a median age of 43 years. Cytogenetic analysis was done in PBLs that were stimulated to grow and cultured for 48 h (Vodicka et al., 2010). About 100 mitoses per person were evaluated to score the frequency of CSAs and CTAs and they were summed up to CAtot (i.e., CSA + CTA = CAtot).

**TABLE 1:** Descriptive attributes of the study cohort and exposure-based distribution.

**Study cohort**			**Covariate effect (*P*-value)^*e*^**
Age (years)	Median	43	0.56
	Range	19–80	
Gender (%)	Females	40.5	0.05
	Males	59.5	
Smoking status (%)	Smokers	66.1	5.55E-05
	Non-smokers	33.9	
Occupational exposure (n)	Small organic compounds	316	2.42E-05
	Heavy metals	6	
	Radiation (pilots)	6	
	Asbestos	19	
	Stone wool	28	
	Glass fibers	18	
	Others^*a*^	214	
No. of individuals with	High CAtot freq^*b*^	342	
	Low CAtot freq	265	
	High CTA freq^*c*^	345	
	Low CTA freq	262	
	High CSA freq^*d*^	321	
	Low CSA freq	286	

*^*a*^Office workers and blood donors who were reported as smokers.*

*^*b*^High CAtot freq = ≥ 2 CAs/100 cells.*

*^*c*^High CTA freq = ≥ 1 CA/100 cells.*

*^*d*^High CSA freq = ≥ 1 CA/100 cells.*

*^*e*^P-value indicates the association of the covariates (age, gender, smoking status, and occupational exposure) with CAs.*

For GWAS genotyping, Illumina HumanOmniExpressExome8v1.3 chip arrays were used and the quality control (QC) criteria were implemented according to the predetermined benchmarks (Niazi et al., 2018, 2019). Samples were included on the basis of successful genotyping ≥95%. Duplicates and related individuals were excluded by identity-by-state (IBS) score. Population outliers determined by the principal component analysis were removed. After prephasing with SHAPEIT v2.12 (Delaneau et al., 2011), imputation was performed using UK10K (Walter et al., 2015) and 1,000 genomes (phase 3, October 2014) (1000 Genomes Project Consortium, Auton et al., 2015) as reference panels with IMPUTE2 v2.3.2 software (Howie et al., 2011). Prior to analysis, SNPs were filtered according to call rate (<95%), Hardy–Weinberg equilibrium (HWE) (*P* < 1.0 × 10^–5^), minor allele frequency (MAF) (<0.05), and imputation quality (Info <0.70).

Association analysis between CA frequency and SNPs in DNA repair genes was conducted using PLINK version 1.90b3.30 (Purcell et al., 2007) using logistic (binary) and linear regression analyses on three phenotypes CAtot, CSAs, and CTAs. For binary logistic regression analysis, individuals were divided into high and low CA frequency groups. For CAtot analysis, individuals with ≥2% CAs were included in the high-frequency group, while for CSAs and CTAs, the threshold for inclusion into the high-frequency group was ≥1% (Dusinska et al., 2004a; Vodicka et al., 2010). The analyses were adjusted for gender, age, smoking status, and occupational exposure. GWAS summary statistics were then used for our gene-based study that included a list of 170 DNA repair genes (Wood et al., 2001, 2005; Friedberg et al., 2006; Lange et al., 2011; Table 2). For these genes, coordinates were extracted from USCS genome browser’s hg19 assembly, which gave a list of genes with chromosome number and transcription start and end position. Genes on the X chromosome were excluded from the analysis as well as those with no match found in NCBI RefSeq list, leaving 153 genes for the analysis (Rosenbloom et al., 2015). On the basis of the gene coordinates, a region including the gene of interest with 100 kb upstream and 100 kb downstream regions was selected, and all the SNPs in this window were analyzed. In total, about 40,000 SNPs from the repair genes’ regions were analyzed, with about 2000 independent loci among them as determined by using PLINK’s linkage disequilibrium-based pruning. These regions were plotted in LocusZoom (Pruim et al., 2010) and SNPs with *P*-value 5 × 10^–3^ or below were further studied for their capacity to influence the functional aspects of the corresponding DNA repair genes. This threshold was set to only select the SNPs above the background level of association in the analysis. *In silico* tools utilized in this analysis were Haploreg, GTex, and RegulomDB 2.0 (Ward and Kellis, 2012; Boyle et al., 2012; GTEx Consortium., 2013). These were used to ascertain linkage disequilibrium (LD) between the SNPs from the same locus identified by different phenotypic analysis as well as location [intergenic, 3′ and 5′ untranslated regions (UTRs)], intronic or expression quantitative trait locus (eQTL, minimal *P*-value of 10^–5^), and effect (synonymous, missense, and non-sense) of the genetic variation. Regulome DB version2.0 provided chromatin state, information about changed motifs, transcription factors, and DNase accessibility.

**TABLE 2:** Total 153 studied genes grouped based on DNA repair type (genes where the SNPs were associated with CAs are in bold letters).

**Base exci sion repair (BER)**	**Other BER and strand break joining factors**	**Poly(ADP-ribose) polym erase (PARP) enzymes that bind to DNA**	**Direct reversal of damage**	**Repair of DNA- -protein crosslinks**	**Mismatch excision repair (MMR)**	**Nucleotide excision repair (NER)**	**Nucleotide excision repair (NER)**	**Homo logous recomb ination (HR)**	**Non-homol ogous end-joining (NHEJ)**	**Fanconi anemia**	**DNA polyme rases (catalytic subunits)**	**Editing and proc essing nucleases**	**Ubiqu itination and mod ification**	**Chromatin structure**	**Genes defective in diseases associated with sensitivity to DNA damaging agents**	**Other identified genes with known or suspected DNA repair function**	**Other conserved DNA damage response genes**
*UNG*	*APEX1 (APE1)*	***PARP1*** *(ADPRT)*	***MGMT***	*TDP1*	*MSH2*	*CDK7*	*XPC*	*RAD51*	*XRCC6 (Ku70)*	*FANCA*	*POLB*	*FEN1 (DNase IV)*	*UBE2B (RAD6B)*	*H2AFX (H2AX)*	*BLM*	*DCLRE1A (SNM1)*	*ATR*
*SMUG1*	*LIG3*	***PARP2*** *(ADPRTL2)*	*ALKBH2 (ABH2)*		*MSH3*	*CCNH*	*RAD23B*	*DMC1*	*XRCC5 (Ku80)*	***FANCC***	*POLG*	*FAN1 (MTMR15)*	*RAD18*	*CHAF1A (CAF1)*	*WRN*	*DCLRE1B (SNM1B)*	***MDC1***
*MBD4*	*XRCC1*	*PARP3 (ADPRTL3)*	*ALKBH3 (DEPC1)*		*MSH6*	*MNAT1*	*RAD23A*	*XRCC2*	*PRKDC*	*BRCA2 (FANCD1)*	*POLD1*	*TREX1 (DNase III)*	*SHPRH*	*SETMAR (METNASE)*	*RECQL4*	*RECQL (RECQ1)*	*RAD1*
*TDG*	*PNKP*				*MLH1*	*ERCC5 (XPG)*	*XPA*	*XRCC3*	*LIG4*	***FANCD2***	*POLE*	*EXO1 (HEX1)*	*HLTF (SMARCA3)*		*ATM*	*RECQL5*	*RAD9A*
*OGG1*	*APLF (C2ORF13)*				***PMS2***	*ERCC1*	*DDB1*	*RAD52*	***XRCC4***	*FANCE*	*PCNA*	*APTX (aprataxin)*	*RNF168*		*TTDN1 (C7orf11)*	*HELQ (HEL308)*	*HUS1*
*MUTYH (MYH)*					*MSH4*	*ERCC4 (XPF)*	*DDB2 (XPE)*	*RAD54L*	*DCLRE1C (Artemis)*	*FANCF*	***REV3L (POLZ)***	*SPO11*	*RNF8*			*RDM1 (RAD52B)*	*RAD17 (RAD24)*
*NTHL1 (NTH1)*					*MSH5*	*LIG1*	*RPA1*	*RAD54B*	*NHEJ1 (XLF, Cernunnos)*	*FANCG (XRCC9)*	*MAD2L2 (REV7)*	*FLJ35220 (ENDOV)*	*RNF4*				*CHEK1*
*MPG*					*MLH3*	*ERCC8 (CSA)*	*RPA2*	*BRCA1*	*NUDT1 (MTH1)*	*FANCI (KIAA1794)*	*POLH*		*UBE2V2 (MMS2)*				*CHEK2*
*NEIL1*					*PMS1*	*ERCC6 (CSB)*	*RPA3*	*RAD50*	*DUT*	***BRIP1 (FANCJ)***	*POLI (RAD30B)*		*UBE2N (UBC13)*				*TP53*
*NEIL2*						*XAB2 (HCNP)*	*ERCC3 (XPB)*	*NBN (NBS1)*	*RRM2B (p53R2)*	*FANCL*	*POLQ*						***TP53BP1*** *(53BP1)*
***NEIL3***						*MMS19L (MMS19)*	*ERCC2 (XPD)*	*RBBP8 (CtIP)*		*FANCM*	*POLK (DINB1)*						*ATRIP*
						*GTF2H1*	*GTF2H3*	*MUS81*		*PALB2 (FANCN)*	*POLL*						*TOPBP1*
						*GTF2H2*	***GTF2H4***			*RAD51C (FANCO)*	*POLM*						*CLK2*
							***GTF2H5 (TTDA)***			*FAAP24 (C19orf40)*	*POLN (POL4P)*						*PER1*

## Results

We identified 14 independent loci associated with CA frequency from six different analyses (two regression models, namely, linear and logistic for each of the three phenotypes, CAtot, CSA, and CTA) below the applied cutoff, *P*-value 5 × 10^–3^; note that the *REV3L* (REV3 like, DNA-directed polymerase zeta catalytic subunit) SNP was detected by both the linear and logistic models in CTA analysis (Table 3). All the SNPs that remained after cutoff *P*-value 5 × 10^–3^ in all phenotypes’ logistic and linear models are given in Supplementary Material. If one would consider the analysis of 153 genes, and assume one association per gene, the Bonferroni type of corrected significance level would have a *P*-value of 3.2 × 10^–4^. SNPs that remained significantly associated with CAs after applying this criterion are indicated in bold in Table 3. Among CAtot associated loci, SNP rs3130780 from logistic regression analysis had a *P*-value that was significant according to such a correction (OR 1.89, 95% CI 1.36–2.64, *P*-value 1.77 × 10^–4^). The SNP is located 1.7 kb 5′ to *GTF2H4* (general transcription factor IIH subunit 4), which belongs to the nucleotide excision repair (NER) pathway. In the same analysis for gene *PARP1* [poly(ADP-ribose polymerase 1], rs1341334 at 1q42.12 with OR 1.56, 95% CI 1.21–2.00, and *P*-value 5.16 × 10^–4^ also came close to the significance threshold. From the linear regression analysis for CAtot, no significant association was identified and the only SNP above the background level was an intronic variant in *MGMT* (O6-methylguanine-DNA methyltransferase) gene. This gene is involved in the direct reversal of DNA damage.

**TABLE 3:** SNP associations with *P*-value ≤ 5 × 10^–3^ from logistic and linear regression analyses of three CA types (CAtot, CTA, and CSA).

**CAtot-logistic**	**DNA repair Gene**	**Type of DNA repair**	**SNP**	**CHR**	**BP**	**A1**	**OR**	**95% CI**	***P***	***In silico***
	*GTF2H4*	Nucleotide excision repair (NER)	**rs3130780**	**6**	**30874308**	**T**	**1.89**	**1.36–2.64**	**1.77E-04**	1.7 kb 5′ of GTF2H4
	*PARP1*	Base excision repair (BER) PARP enzymes	rs1341334	1	226605024	G	1.56	1.21–2.00	5.16E-04	9.2 kb 5′ of PARP1/eQTL

**CAtot-linear**	**DNA repair Gene**	**Type of DNA repair**	**SNP**	**CHR**	**BP**	**A1**	**Beta**	**95% CI**	***P***	***In silico***

	*MGMT*	Direct reversal of DNA damage	rs12247555	10	131370520	C	0.09	0.03–0.15	2.78E-03	Intronic/eQTL

**CTA-logistic**	**DNA repair Gene**	**Type of DNA repair**	**SNP**	**CHR**	**BP**	**A1**	**OR**	**95% CI**	**P**	***In silico***

	*NEIL3*	Base excision repair (BER)	rs10009807	4	178229925	A	0.69	0.54–0.89	4.62E-03	1.1 kb 5′ of NEIL3/histone marks
	*REV3L*	DNA polymerases (catalytic subunits)	rs7742724	6	111839019	A	1.63	1.23–2.16	6.42E-04	eQTL
	*BRIP1*	Fanconi anemia	rs17542001	17	59915590	C	0.64	0.48–0.85	1.86E-03	Intronic/eQTL

**CTA-linear**	**DNA repair Gene**	**Type of DNA repair**	**SNP**	**CHR**	**BP**	**A1**	**Beta**	**95% CI**	***P***	***In silico***

	*FANCC*	Fanconi anemia	rs13292454	9	97995075	A	0.16	0.05–0.28	3.93E-03	Intronic
	*MDC1*	Conserved DNA damage response genes	rs3094090	6	30669956	C	0.19	0.07–0.32	2.33E-03	Intronic
	*REV3L*	DNA polymerases (catalytic subunits)	**rs7742724**	**6**	**111839019**	**A**	**0.16**	**0.09–0.24**	**2.47E-05**	eQTL
	*XRCC4*	Non-homologous end-joining (NHEJ)	rs301286	5	82602955	C	−0.14	(−0.22)–(−0.05)	1.42E-03	Intronic/histone marks
	*TP53BP1*	Conserved DNA damage response genes	rs28702649	15	43648629	T	0.12	0.05–0.19	1.31E-03	eQTL

**CSA-logistic**	**DNA repair Gene**	**Type of DNA repair**	**SNP**	**CHR**	**BP**	**A1**	**OR**	**95% CI**	***P***	***In silico***

	*GTF2H5*	Nucleotide excision repair (NER)	**rs1744178**	**6**	**158496856**	**T**	**1.74**	**1.31–2.31**	**1.50E-04**	eQTL
	*PARP2*	Base excision repair (BER) PARP enzymes	rs2318861	14	20758949	G	0.54	0.38–0.78	9.85E-04	eQTL

**CSA-linear**	**DNA repair Gene**	**Type of DNA repair**	**SNP**	**CHR**	**BP**	**A1**	**Beta**	**95% CI**	**P**	***In silico***

	*FANCD2*	Fanconi anemia	rs61429272	3	10037320	C	0.15	0.05–0.24	4.13E-03	eQTL
	*PMS2*	Mismatch excision repair (MMR)	rs12702464	7	6041506	C	−0.13	(−0.21)–(−0.04)	4.64E-03	Intronic/eQTL

*ORs (in logistic regression analysis), Beta values (in linear regression analysis), and their corresponding P-values and in silico predictions are shown. SNP associations that survived Bonferroni correction for multiple testing are marked in bold. SNP single nucleotide polymorphism; CHR chromosome; OR odds ratios; A1 The allele for which beta and OR are calculated.*

A SNP marking the gene *REV3L* was found to be associated with the CTA phenotype in both linear and binary logistic regression models. This variant, rs7742724, exhibited a notable association in the linear model with β 0.16, 95% CI 0.09–0.24, and *P*-value 2.47 × 10^–5^ and a similar tendency in the binary model but with an elevated *P*-value of 6.42 × 10^–4^. The other variants from the CTA analysis had *P*-values ranging from 1.31 × 10^–3^ to 4.62 × 10^–3^. These included intronic SNPs in the genes *BRIP1* (BRCA1 interacting protein C-terminal helicase 1), *FANCC* (Fanconi anemia complementation group C), *MDC1* (mediator of DNA damage checkpoint 1), and *XRCC4* (X-ray repair cross-complementing protein 4) as well as in *TP53BP1* (TP53-binding protein 1). *BRIP1* and *FANCC* belong to the Fanconi anemia pathway while *MDC1* and *TP53BP1* are conserved DNA damage response genes. *XRCC4* is an NHEJ gene. A variant rs10009807 located at 1.1 kb 5′ to *NEIL3* (nei like DNA glycosylase 3), which is a base excision repair (BER) pathway gene, was also among the associations identified from the CTA analysis.

The CSA group presented a total of four associations from both models; SNP at 6q25.3 and eQTL to *GTF2H5* (general transcription factor IIH subunit 5) was the best candidate with OR 1.74, 95% CI 1.31–2.31, and *P*-value 1.50 × 10^–4^. *GTF2H5* is a member of the NER pathway. rs2318861, an eQTL SNP, for *PARP2* [poly(ADP-ribose) polymerase 2], which ADP-ribosylates DNA by acting on terminal phosphates at DNA strand breaks, had a *P*-value of 9.85 × 10^–4^. In the linear regression analysis for CSAs, two associations included a Fanconi anemia gene *FANCD2* (Fanconi anemia complementation group D2) and an intronic variant for mismatch repair (MMR) pathway gene *PMS2* (PMS1 homolog 2, mismatch repair system component).

In Table 4, the candidate SNPs were annotated using RegulomeDB and GTex; some eQTL data were also retrieved using Haploreg. SNPs linked to genes *PARP1, NEIL3*, *FANCC, XRCC4*, and *FANCD2* show DNase accessibility in blood and all the selected variants were located in either the region of strong transcription or in transcription start sites (TSSs) and enhancers in blood and many other tissues. The eQTLs summarized in Table 4 each target the linked DNA repair gene. The SNP linked to *PARP1* was a strong eQTL in cultured fibroblasts at 9 × 10^–23^, and the one linked to the *MGMT* gene was a strong eQTL in the whole blood at 1.4 × 10^–33^. The SNPs associated with *MDC1*, *GTF2H5, PARP2*, and *FANCD2* were eQTLs in the whole blood/cultured fibroblasts. The SNP for *PMS2* was an eQTL in the aorta, whereas those linked to genes *REV3L* and *BRIP1* were eQTLs in the brain and the tibial nerve, respectively.

**TABLE 4:** Regulome DB 2.0/GTex *in silico* analysis of associated variants.

**Chr**	**SNP**	**Gene**	**Accessibility (DNase and FAIRE) in tissues/cell types**	**Chromatin state**	**Motifs**	**eQTL**			**ChIP**
				**Strong transcription (no. of tissues and cell lines)**		**Tissue**	Normalized effect size*	***P*-value**	
6	rs3130780	*GTF2H4*	−	Blood + 102	BCL6, MEF2A	−	−	−	ZNF792, MIXL1
1	rs1341334	*PARP1*	Blood (K562) + 9 other tissues	Enhancer in blood	BCL6, NANOG	Cultured fibroblasts	−0.31	9.00E-23	ZFX,ZNF770
10	rs12247555	*MGMT*	−	Blood + 16	−	Whole blood	−0.29	1.40E-33	−
4	rs10009807	*NEIL3*	Blood, B cells, T cells	Enhancer in blood + 30	−	−	−	−	POLR2A
17	rs17542001	*BRIP1*	−	Blood + 62	−	Nerve–tibial	−0.41	7.30E-15	−
9	rs13292454	*FANCC*	Blood + 3 others	Blood + 48	FOXJ3	−	−	−	−
6	rs3094090	*MDC1*	Tibial nerve	Blood + 123	IRF3	Whole blood	0.13	6.40E-06	ZNF664
6	rs7742724	*REV3L*	Mammary glands	Blood + 23	−	Brain–Cerebellum	0.28	1.40E-06	SMARCA4 ^#^
5	rs301286	*XRCC4*	Blood + 6 others	Active TSS in blood + 6	−	−	−	−	STAT5A^#^, STAT3^#^, TBP^#^ + 10
15	rs28702649	*TP53BP1*	Placenta, H9, OCI-LY7	Blood + 109	SOX1	Cultured fibroblasts	−0.13	4.10E-08	CTCF^#^, RAD21, ZBTB33^#^
6	rs1744178	*GTF2H5*	H7-hESC, Lower leg	Blood + 118	−	*Whole blood	*	2.8783E-17	−
14	rs2318861	*PARP2*	−	Blood + 124	−	*Whole blood	*	6.61974E-07	ZBTB40
3	rs61429272	*FANCD2*	Naïve B cell + 9 other tissues	Blood + 99	−	Whole blood	−0.17	2.70E-09	−
7	rs12702464	*PMS2*	−	Blood + 124	−	Artery–aorta	−0.44	4.50E-12	−

*eQTL values *from* Westra et al. (2013).

*^#^In blood.*

## Discussion

Genetic variation can be the cause of inter-individual differences in susceptibility to CAs and susceptibility to cancer (Vodicka et al., 2004a). In a previous analysis of 11 DNA repair genes in mixed population of occupationally exposed and unexposed individuals, associations with CAs were found with *XPD* and *RAD54L* polymorphisms (Vodicka et al., 2015). In the present study, SNPs from a total of 153 DNA repair genes were tested on an exposed population of 607 individuals. It can be expected that DNA repair is more critical in persons exposed to high apparent exposure vs. background environmental exposure and the distribution of CAs has been shown to be skewed to higher damage levels in the exposed population (Niazi et al., 2019). This population difference together with a more stringent significance threshold in the present study might be the reason for the different outcomes of these two studies. While CSAs and CTAs are assumed to be independent markers of damage, arising at different phases of the cell cycle, CAtot is a composite measure as the sum of CSAs and CTAs. For the presentation of the results, we selected an arbitrary cutoff level of 5 × 10^–3^, which appeared to be stringent as only 14 SNPs passed the threshold and, with one exception, these results from the linear and logistic models were different. We considered the Bonferroni type of adjusted *P*-value as 3.2 × 10^–4^ based on the 153 genes tested; even though many more independent LD regions were considered, the sample size for rarer SNPs afforded a limited power (with a MAF of 10%, only six homozygous variants were to be expected). Credibility to the findings is supported by the chromatin state and eQTL data. All SNPs were located at a site of strong transcription, enhancer, or TSS, and five SNPs were located at DNase-accessible sites in blood. Ten of the 14 candidate SNPs influenced the expression of the target DNA repair gene, and for 5, the data were obtained from whole blood.

Most positive associations at *P*-value below 5 × 10^–3^ were found for CTAs (*N* = 7), followed by four for CSAs and three for CAtot. SNPs for the NER pathway emerged two times, for BER and Fanconi anemia repair pathways three times, while SNPs in other pathways were unique. For the BER pathway, the implicated SNPs were eQTLs to the target genes *PARP1* and *PARP2*, two homologs encoding chromatin-associated enzymes, poly(ADP-ribosyl)transferases, which modify various nuclear proteins by poly(ADP-ribosyl)ation with multiple downstream regulatory functions (Azarm and Smith, 2020). PARP1 may be associated with xeroderma pigmentosum, complementation group A through interactions with XPA, and the related susceptibility to skin cancer. PARP1 is involved in the synthesis of telomere C-strand (Azarm and Smith, 2020). PARP2 has partially overlapping biochemical functions with PARP1. PARP1 and PARP2 function in both single- and double-strand DNA repair, and they have an important role in maintaining genome stability through diverse mechanisms. PARP inhibitors are being used as anticancer agents in *BRCA1/2* mutated cancers (Boussios et al., 2020).

The NER pathway genes *GTF2H4* and *GTF2H5* encode different subunits of general transcription factor IIH and both associations were highly significant (Rimel and Taatjes, 2018; Hill and Theos, 2019; Kolesnikova et al., 2019). The proteins share structural and functional homology and they are associated with NER enzymes XPB and XPD (Kolesnikova et al., 2019). Syndromes associated with *GTF2H4/5* include xeroderma pigmentosum, complementation groups B and D, Cockayne syndrome, and trichothiodystrophy, all of which are characterized by extreme sensitivity to ultraviolet radiation and development of other sun-related problems such as excessive freckling and skin cancer. These syndromes as well as Fanconi anemia germline mutations display genomic instability and CAs (Chan and Ngeow, 2017; Hill and Theos, 2019). The related SNP targeted *GTF2H5* as an eQTL.

In summary, the present study on DNA repair gene polymorphisms in a healthy population with occupational and personal genotoxic exposures revealed SNP associations with CA frequency at the *P*-value level of 5 × 10^–3^ within 14 different genes, many of which with key roles in maintaining genomic integrity and thus plausibly associated with mechanisms leading to CAs. More than half of the implicated SNPs were eQTLs to the target DNA repair genes. Although the recent interest in measuring random CAs has decreased because of cumbersome techniques, the present results suggest that the results may have understandable mechanistic links. If the current techniques cannot be improved, there will be a need to provide alternative approaches for measuring individual sensitivity to genotoxic exposure that may lead to increased risk of cancer.

## Data Availability Statement

All the relevant data presented in the study is given in Supplementary Material, further inquiries can be directed to the corresponding author/s.

## Ethics Statement

The studies involving human participants were reviewed and approved by the Ethics Committees of the Slovak Medical University, the Jessenius Faculty of Medicine, the Comenius University Bratislava, the Institute for Clinical and Experimental Medicine in Slovakia and the Thomayer Hospital and the General University Hospital in the Czech Republic. The patients/participants provided their written informed consent to participate in this study.

## Author Contributions

AF, KH, YN designed the study. HT and YN analyzed the data. YN and BS performed the genotyping. PV, LV, LM, MD, and BS provided the samples. SV, MK, VV, KV, AK, and MS collected subject information and performed cytogenetic analysis. PH and MN were responsible for the GWAS. AF, KH, PV, LV, MD, and BS critically revised the manuscript. YN wrote the first draft. KH and AF supervised the study. All authors contributed to manuscript revision, read, and approved the submitted version.

## Conflict of Interest

HT was an employee at GeneWerk GmbH, which had no role in the design of the study; in the collection, analyses, or interpretation of data; in the writing of the manuscript, or in the decision to publish the results. The remaining authors declare that the research was conducted in the absence of any commercial or financial relationships that could be construed as a potential conflict of interest.
